# Epilepsy’s effect on cardiac rhythm and the autonomic nervous system

**DOI:** 10.1590/1806-9282.20230742

**Published:** 2024-01-22

**Authors:** Tulin Gesoglu Demir, Fatih Gungoren, Ozlem Uzunkaya Ethemoglu, Dilek Agircan

**Affiliations:** 1 Harran University, Faculty of Medicine, Department of Neurology – Şanlıurfa, Turkey.; 2 Medical Park Florya Hospital, Department of Cardiology – İstanbul, Turkey.

**Keywords:** Electrocardiography, Epilepsy, Electrodermal response

## Abstract

**OBJECTIVE:**

Sudden unexpected death in epilepsy is the most common cause of death in young patients with epilepsy. The aim of this study was to evaluate changes in interictal electrocardiogram parameters and sympathetic skin responses as markers of autonomic dysfunction in patients with epilepsy and to determine their effects on the type and duration of epilepsy, frequency of seizures, and responses to treatment.

**METHODS:**

A total of 97 patients with epilepsy and 94 healthy controls were recruited. We recorded their clinical and demographic characteristics and analyzed sympathetic skin response latency and amplitude, electrocardiogram recordings, and seven cardiac rhythm parameters: P-wave duration, PR segment, QRS duration, QT interval, QT interval distribution, Tpe duration, and Tpe/QT interval ratio.

**RESULTS:**

P-wave durations, T-wave durations, QT and QT interval durations, and Tpe and sympathetic skin response latency were significantly longer among patients with epilepsy than the controls, and their heart rate was significantly lower. However, sympathetic skin response latency and heart rate were negatively correlated, and T-wave duration, QT duration, QT interval duration, and Tpe were positively correlated.

**CONCLUSION:**

Our results from interictal electrocardiograms indicate clinically significant arrhythmias among patients with epilepsy and the correlation of such arrhythmias with sympathetic skin responses. Thus, noninvasive tests that evaluate the autonomic system should be used to predict the risk of sudden unexpected death in epilepsy among patients with epilepsy.

## INTRODUCTION

Individuals with epilepsy, facing a higher risk of death than the general population, may suffer sudden unexpected death in epilepsy (SUDEP), the most common direct cause of premature death associated with the condition^1^. It is likely not an isolated disorder of a single system but instead a series of events that affect autonomic and cardiorespiratory regulation^2^.

In particular, previous findings have suggested that an increased QT dispersion (QTd) prompts mortal ventricular arrhythmias and sudden cardiac death^3^, while both the Tpe interval and increased Tpe/corrected QT (Tpe/QTc) ratio are associated with life-threatening ventricular arrhythmias^4,5^. However, literature examining ECG parameters such as the Tpe interval and Tpe/QTc ratio in the interictal period, despite their direct relationship with mortal arrhythmias, remains sorely limited. Based on this, seizures have been shown to trigger the activity of the sympathetic nervous system and increase both heart rate (HR) and blood pressure, and the system’s over activity has been associated with ventricular tachyarrhythmias and sudden death^6^.

In our study, we therefore aimed to evaluate the interictal changes in ECGs and SSR activities among patients with epilepsy and to evaluate the relationship of those variables with seizure semiology, including the type and duration of epilepsy and the frequency of seizures.

## METHODS

Our prospective case-control study was approved by the Ethics Committee of Harran University Faculty of Medicine (18.10.2021, HRU/21.18.31), and written informed consent was obtained from all participants. For a patient group, we recruited 97 patients more than 18 years old, who were diagnosed with epilepsy according to the International Epilepsy League’s criteria, and who visited the epilepsy outpatient clinic between January and June 2022. Their data regarding clinical semiology, electroencephalography, and neuroimaging were recorded. For a control group, we recruited 94 age- and gender-matched healthy volunteers who visited our neurology outpatient clinic. Their ECGs and SSRs were checked for differential diagnosis, but the individuals were found to be normal and no associated disease was found. Any prospective participants currently taking drugs that can affect the autonomous nervous system (ANS) functions or who had a disease that can affect the ANS function were excluded from the sample, as were those staking drugs that can affect ECG parameters were excluded^7^.

After detailed neurological examinations, SSRs were measured in all participants grouped in a semi-dark, quiet room maintained at 22–24°C and with their skin temperature no lower than 32°C. The participants were in the resting position, and after at least 4 h, they had consumed any nicotine- or caffeine-containing substances (e.g., cigarettes, tea, and coffee). To gather SSRs, we placed an active Ag/AgCl electrode on each patient’s palm and the reference electrode on the back of their hand. Meanwhile, the earth electrode was placed in the midline of the frontal region of the head. During recording, the filter setting of the device was 0.5 Hz–1 kHz, the sensitivity was 500 μV, and the sweep interval was 0.2 s.

Before ECGs, the participants’ blood pressure (BP) was measured, and their systolic and diastolic BPs were recorded. ECGs were recorded in a 12-channel form at a standard of 10 mm/mV and a speed of 25 mm/s. ECGs were evaluated by a cardiologist blind to the patient versus control groups. All QRS complexes were examined for ectopic beats, and the ones with such beats were discarded. In the ECGs, HR, P-wave duration, PR interval, PR segment, QRS duration, and T-wave duration were evaluated, as was the QT interval in all leads. In contrast, the QT interval was measured manually. In addition, QTd was calculated by determining the difference between the maximum QT value (QT-max) and the minimum QT value (QT-min), while corrected QT was calculated using the Bazett formula (i.e., QTc=QT/√RR). In the precordial leads, Tpe was determined by measuring the time between the peak of the T wave (i.e., peak for the positive T wave and the deepest point for the negative T wave) and the end of the wave using the tangent method. The Tpe/QT-to-Tpe/QTc ratio was calculated using precordial lead measurements, which ensured that both parameters were from the same lead.

## RESULTS

The mean ages of the patient and control groups were 30.41±10.13 and 32.82±11.06, respectively. Table 1 presents the characteristics of seizures and patients with epilepsy.

**Table 1.** Characteristics of seizures and patients with epilepsy.

		Controlled epilepsy group, n (%)	Resistant epilepsy group, n (%)	p
Seizure type	Focal	18 (23.7%)	10 (47.6%)	0.235
	Generalized	50 (65.8%)	7 (33.3%)	
	FBTCS	8 (10.5%)	4 (19%)	
Etiology	Structural/metabolic	12 (15.8%)	3 (14.3%)	0.845
	Genetic	4 (5.3%)	2 (9.5%)	
	Unknown	60 (78.9%)	16 (76.2%)	
Family history	Yes	10 (13.2%)	3 (14.3%)	0.894
	No	66 (86.8%)	18 (85.7%)	
EEG	Normal	33 (43.4%)	12 (57.1%)	0.275
	Focal	11 (14.5%)	3 (14.3%)	
	Generalized	24 (31.6%)	4 (19%)	
	Slow	8 (10.5%)	2 (9.5%)	
MRI	Normal	51 (67.1%)	14 (66.7%)	0.979
	Lesional	14 (18.4%)	4 (19%)	
	Nonspecific	11 (14.5%)	3 (14.3)	
Therapy	Monotherapy	54 (71.1%)	2 (9.5%)	**0.000**
	Polytherapy	22 (28.9%)	19 (90.5%)	
Therapy type	Na channel blockers (+)	48 (63.2%)	28 (36.8%)	**0.001**
	Na channel blockers (-)	21 (100%)	0	
Seizure frequency		0.98±1.07	4.33±2.03	**0.000**
Disease duration		9.42±6.06	15.09±8.74	**0.010**
Disease onset age		19.53±9.15	20.57±12.52	0.727

EEG: electroencephalography; MRI: magnetic resonance imaging; FBTCS: focal to bilateral tonic-clonic seizure. Bold indicates statistically significant p-value.

Concerning ECG parameters, the HR of the patients was significantly lower than the control group (p=0.019), while their T-wave duration, QT duration, and Tpe were significantly longer (p==0.000, p==0.000, and p==0.004, respectively) (Table 2). Also, as shown in Table 2, the P-wave duration was also significantly longer among patients versus controls (p==0.007). The P-wave duration was longer among patients with resistant epilepsy versus the ones with controlled epilepsy (p==0.001). Although the mean QTc was significantly longer among patients than controls (p==0.000), it was significantly shorter among patients with resistant epilepsy than among ones with controlled epilepsy (p==0.011). There was no other statistically significant difference (p>0.05) in the parameters between the resistant and controlled epilepsy groups (Table 3).

**Table 2.** Characteristics of electroencephalography and sympathetic skin response parameters in patients with epilepsy and control groups.

	Patient	Control	p
SSR latency	1607.30±232.36	1306.90±112.79	**0.000**
SSR amplitude	2.42±1.94	2.56±1.73	0.583
Heart rate	74.64±13.54	79.59±15.37	**0.019**
P-wave duration	52.78±14.84	47.65±10.61	**0.007**
PR interval	118.76±21.32	118.93±18.80	0.953
PR segment	66.28±20.78	71.80±18.66	0.055
QRS duration	64.53±12.16	62.97±6.85	0.279
T-wave duration	125.56±25.85	109.68±18.16	**0.000**
QT duration	350.61±33.99	323.93±22.44	**0.000**
QTc duration	393.40±31.83	365.74±24.25	**0.000**
Tpe	57.01±14.15	51.91±9.86	**0.004**
Systolic BP	108.25±16.58	111.48±13.43	0.141
Diastolic BP	69.58±9.78	69.68±10.41	0.949
Tpe/QT ratio	0.16±0.03	0.16±0.02	0.678
Tpe/QTc ratio	0.14±0.03	0.14±0.03	0.603

ECG: electrocardiogram; SSR: sympathetic skin response; BP: blood pressure. Bold indicates statistically significant p-value.

**Table 3.** Characteristics of electroencephalography and sympathetic skin response parameters in patients with epilepsy and control groups.

	Controlled epilepsy group	Resistant epilepsy group	p
SSR latency	1605.51±238.05	1613.80±215.86	0.886
SSR amplitude	2.61±2.09	1.72±1.02	0.064
Heart rate	75.85±13.39	70.28±13.49	0.096
P-wave duration	50.13±13.51	62.38±15.78	**0.001**
PR interval	117.36±21.99	123.80±18.29	0.222
PR segment	67.50±21.23	61.90±18.87	0.277
QRS duration	63.94±11.32	66.66±14.94	0.367
T-wave duration	124.60±24.62	129.04±30.31	0.489
QT duration	350.13±33.96	352.38±34.91	0.790
QTc duration	397.69±30.27	377.85±33.22	**0.011**
Tpe	57.10±14.31	56.66±13.90	0.901
Systolic BP	108.96±18.15	105.71±8.70	0.430
Diastolic BP	70.13±9.99	67.61±8.89	0.300
Tpe/QT ratio	0.16±0.03	0.16±0.03	0.787
Tpe/QTc ratio	0.14±0.03	0.15±0.03	0.473

ECG: electrocardiogram; SSR: sympathetic skin response; BP: blood pressure. Bold indicates statistically significant p-value.

There was no significant difference in ECG parameters and SSRs between patients using monotherapy and patients using polytherapy, or between patients using Na channel blockers and patients not using them (p>0.05).

The SSR latency of patients was significantly higher than that of the controls (p=0.000), whereas their SSR amplitude did not differ (Table 2). Patients with controlled versus resistant epilepsy did not differ in SSR amplitude or latency (p>0.05) (Table 3).

A significant positive correlation was found between disease duration and PR distance (p==0.002, r=0.312), while there was a negative correlation between SSR latency and HR (p=0.008, r=-0.192). There was a positive correlation between T-wave duration, QT duration, QTc duration, and Tpe (p==0.000, r=0.300; p==0.000, r=0.351; p==0.001, r=0.243; p==0.020, r=0.169, respectively).

## DISCUSSION

Researchers have shown heightened interest in autonomic dysfunction as a potential biomarker of SUDEP. Although the mechanisms underlying SUDEP remain unclear, various animal and human studies have shown that interictal sympathetic activation and variable vagal tone may be responsible. It is also known that autonomic and cardiac dysfunctions worsen over time among patients with epilepsy at high risk of SUDEP^8^.

Epilepsy can affect ANS functions by causing changes in central nervous system functions, both with seizures in the ictal period and epileptiform discharges in the interictal period^9^. Interictal autonomic modulations, especially cardiac dysfunctions, are believed to be responsible for SUDEP^10^.

Although seizures often cause temporary changes in HR and BP, recurrent seizures among patients with epilepsy may affect resting BP and HR, with an increase in interictal sympathetic tone. In our study, the patients’ HR was significantly lower than the controls’ HR, and no significant difference in HR emerged between the resistant and controlled epilepsy groups. There was also no significant difference between the patient and control groups in terms of systolic and diastolic BPs. In another case-control study in which ECG parameters were evaluated, no significant difference surfaced in HR between patients with epilepsy in the interictal period and the control group^11^. Moreover, in their research evaluating HR and BP among patients with epilepsy, Nei et al., found similar results between epilepsy and control groups and reported a trend toward higher diastolic BP and more stable HR among patients with epilepsy who died from SUDEP^12^.

The P-wave duration and PR interval represent atrial electrical activity. In our study, the P-wave duration was significantly longer among the patients than the controls and among patients with resistant epilepsy than controlled epilepsy. Although the PR distance did not differ significantly between the patient and control groups, a significant positive correlation was found between it and disease duration. In De Sousa et al.’s study, the P-wave duration and PR interval were significantly longer among patients with epilepsy than controls^11^. According to the study that correlates the current findings with recurrent seizures and duration of epilepsy, the difference in PR distance in our patients can be explained by the shorter mean duration of epilepsy in our sample.

In ECGs, various ventricular repolarization markers, including QT interval^13^, QTc^14^, Tpe^15^, Tpe/QT ratio, Tpe, and QTc, have been used to predict the prevalence of cardiac arrhythmias.

In studies on ventricular repolarization, it was reported that the Tpe interval and Tpe/QT ratio increased in patients with severe coronary artery disease evaluated with the SYNTAX score^16^. In another study, it was stated that these parameters were not significantly associated with the severity of CAD^17^.

Similar to De Sousa et al.’s study^11^, Dagar et al.^18^ found that QT and QTc intervals were higher in the interictal period among patients with epileptic seizures than among healthy individuals. In our study, although the QTc interval was significantly longer among patients than controls, it was significantly shorter among patients with resistant epilepsy than ones with controlled epilepsy.

The Tpe interval, indicating transmural cardiac repolarization, is an important indicator of the risk of ventricular arrhythmias and has been shown to be a more accurate predictor of cardiac arrhythmias than QTc^13^. A meta-analysis comprising 155,856 patients showed that a prolonged Tpe interval is an important determinant of arrhythmia and mortality^14^. In a study evaluating the Tpe interval among patients with epilepsy, no significant difference emerged between patients with epilepsy and controls^18^; however, in our study, the patients’ Tpe interval was significantly longer than the controls.

The ratio between Tpe and QT, as a new marker of cardiac arrhythmias, is claimed to be more accurate than other markers^13^. In our study, no significant difference in Tpe/QT and Tpe/QTc ratios appeared between the patient and control groups. In another study conducted in an emergency room, the Tpe/QTc ratio was significantly prolonged among patients with epilepsy compared with the controls, but no significant difference arose between patients with first-time seizures and patients with epilepsy^18^. Differences may be due to sample sizes, the duration of illness, and/or antiseizure drugs (ASDs) used.

Neuronal recordings and immunostaining demonstrate the presence of parasympathetic and several sympathetic efferents within the intrinsic cardiac plexus, as well as afferents that respond to different mechanical and chemical stimuli. Afferent-mediated activation of neurohumoral systems increases the sympathetic impulse and decreases the vagal tone. This maintains the cardiac output in the short term, but maintaining the cardiac output in this way causes increased myocardial oxygen demand and excessive Ca^2+^ overload in cardiomyocytes. Chronic abnormal cardiac afferent signaling causes persistent sympathetic activity and cardiovagal loss, increasing the likelihood of sudden death from heart pumping failure and arrhythmia^19^.

In the study, a negative correlation was found between SSR latency and HR, while a positive correlation was found between T-wave duration, QT duration, QTc duration and Tpe, which are ECG parameters that are indicators of ventricular repolarization. Accordingly, ECG and SSR may be useful in the follow-up of patients with epilepsy in terms of the risk of SUDEP.

In our study, in which we evaluated sympathetic sensitivity from autonomic findings, the CSR latency of patients with epilepsy in the interictal period was significantly longer than in the control group. However, SSR amplitudes did not differ significantly between the two groups. Meanwhile, in the resistant and controlled epilepsy groups, no significant difference arose in SSR latency or amplitude. In a study comparing 50 patients with epilepsy in the interictal period with controls, Drake et al., found that the patients had significantly longer SSR latencies and significantly higher SSR amplitudes^20^. In Atalar et al.’s study, while SSR amplitudes were significantly higher among patients with epilepsy than controls, the authors did not detect a significant difference between latencies^21^. By comparison, in the Berilgen et al.’s study, SSR latencies were longer in the partial epilepsy group than in the control group; however, they did not report differences in SSR amplitudes between patients and controls^22^. Although a high SSR amplitude may indicate increased sympathetic sensitivity^23^, the SSR latency length is arguably a more objective parameter because it is less affected by habituation^1^.

Various ASDs can cause abnormalities in the cardiac conduction system. Although numerous case–control studies have suggested that ASDs are a strong risk factor for SUDEP, particularly when given in polytherapy or when more than two changes in ASD occur per year, and data in the literature do not confirm that hypothesis^24^. Some ASDs are known to affect cardiac conduction by blocking voltage-gated Na channels^25^. In our study, no significant difference emerged in ECG parameters and SSRs between patients using monotherapy and patients using polytherapy or between patients using and patients not using Na channel blockers.

## CONCLUSION

Patients with epilepsy may have a higher risk of life-threatening malignant arrhythmias than the nonepileptic population. In our study, an increased risk of arrhythmia among patients with epilepsy, the correlation of that risk with SSR, and the relationship between cardiac arrhythmias and the autonomic system were found. Thus, even if patients do not have autonomic symptoms during follow-up, they should be carefully evaluated in that respect.

## LIMITATIONS

Among our study’s limitations, the ECG data were cross-sectional and obtained in short term, and the long-term ECG monitoring of the patients was not performed.

## ETHICAL CONSIDERATIONS AND DISCLOSURE

The study was approved by the Institutional Ethical Board of Harran University Faculty of Medicine (Decision no: HRU/21.18.31; Date: 18.10.2021).
